# Negative Pressure Wound Therapy with Instillation and Dwell Time in the Surgical Management of Severe Hidradenitis Suppurativa

**DOI:** 10.7759/cureus.3319

**Published:** 2018-09-17

**Authors:** Shealinna Ge, Hakan Orbay, Ronald P Silverman, Yvonne M Rasko

**Affiliations:** 1 Plastic and Reconstructive Surgery, University of Maryland School of Medicine, Baltimore, USA; 2 Surgery, University of Maryland School of Medicine, Baltimore, USA; 3 Plastic and Reconstructive Surgery, University of Maryland School of Medicine, Baltimore , USA

**Keywords:** wound healing, hidradenitis suppurativa, negative pressure wound therapy with instillation and dwell, npwti-d, skin grafting

## Abstract

Background: Hidradenitis suppurativa (HS) is a physically debilitating disease that greatly impairs the quality of life of affected individuals. Advanced disease is often difficult to treat with topical and systemic therapies. Surgical resection of diseased skin has become paramount in HS management but proposes challenges of wound care and closure.

Methods: Four patients with a total of 12 complex wounds were treated over a three year period. All of the patients were males between the ages of 28 and 61 years. The lesions were located on the buttocks (n=5), chest (n=1), perianal (n=2), perineal (n=2), and axillary regions (n=2). A protocol of wide local excision, followed by negative pressure wound therapy with instillation and dwell time (NPWTi-d) to decrease bioburden and promote angiogenesis of the exposed base, and subsequent skin grafting was used. Patients remained hospitalized between procedures.

Results: The original wound area ranged from 210-540 cm^2^. Skin grafts of comparable sizes were taken from donor sites. The average duration of NPWTi-d placement was 3.5 days and the average time from excision to wound coverage was 4.3 days. The percent of graft uptake ranged from 70%-90%. All patients were resolved of their local disease with no complications.

Conclusions: Surgical management of HS can be complicated by difficult closures. This case series demonstrates that wide local excision followed by NPWTi-d and skin grafting is able to achieve local resolution of disease in HS patients who have failed multiple minimally invasive therapies.

## Introduction

Hidradenitis suppurativa (HS) is a chronic inflammatory disease that selectively impacts apocrine gland-bearing skin and most frequently involves the skin of intertriginous areas, such as the axillary, groin, anogenital, and inframammary regions. HS is characterized by recurrent and painful flares that significantly impair the quality of life and negatively impact the social, psychological, and sexual health of affected individuals [1-2]. Presentations of HS range from mild, such as subcutaneous nodules with moderate inflammation that regress without draining, to severe, where patients experience painful, diffuse abscesses that eventually coalesce to form sinus tracts containing foul-smelling, purulent, or serosanguinous drainage. Severe presentations can eventually lead to subsequent scarring and disfigurement. Early stage HS may resemble other disorders, often delaying diagnosis and treatment [3]. The true prevalence of HS is unknown, but estimates indicate that as high as 4% of the population may be affected, with the highest incidence in the second and third decades of life. Women are three times more likely to develop HS than men, and African Americans are more likely to be affected than other ethnicities [2].

Clinically, the Hurley and Sartorius systems have been used to stage HS lesions. The Hurley staging system is divided into three stages based on the presence and extent of disease. Stage I is comprised of single episodes of abscess growth without sinus tract formation, whereas stage II is characterized by recurrent episodes of abscess formation with sinus tract development and scarring. Finally, stage III encompasses diffuse abscess formation with the development of interconnected sinus tracts across entire regions of tissue and widespread scarring [4]. The Sartorius scoring system is more complex and incorporates the anatomical distribution, number, and type of lesions, as well as the distance and presence of normal tissue between lesions [5].

Negative pressure wound therapy (NPWT), most commonly used in the management of open wounds, has been adapted to treat Hurley stage II and III HS in conjunction with surgical debridement. NPWT employs a vacuum controlled system to create a subatmospheric layer of pressure that is distributed symmetrically over a wound through an open cell foam dressing. NPWT is simple yet effective for the promotion of granulation tissue formation in acute and chronic wounds, and has been shown to reduce wound contamination and promote angiogenesis [6]. NPWT with instillation and dwell time (NPWTi-d) is a new form of NPWT initially used for the treatment of infected orthopedic prosthesis [7-8]. This system intermittently pauses NPWT to instill an antiseptic solution on the wound and allows the solution to stay on the wound for a predetermined period of time. NPWT is resumed at the end of this dwell phase.

In this study, we report a series of patients with severe HS complicated by wound contamination treated with combined wide surgical resection and NPWTi-d. Our aim is to introduce a new treatment option in the management of this challenging disease.

## Materials and methods

Participants included four male African American patients, aged 28-61 years, body mass index (BMI) 19.3-36.7, with a total of 12 complex and contaminated wounds due to longstanding Hurley stage III HS. Five on the buttocks, one on the chest, and two in each of the perianal, perineal, and axillary regions. All patients developed significant sinus tracts, abscesses, and fistulization. All patients had failed multiple courses of topical and systemic therapies, requiring definitive treatment with wide surgical excision and debridement.

**Figure 1:**
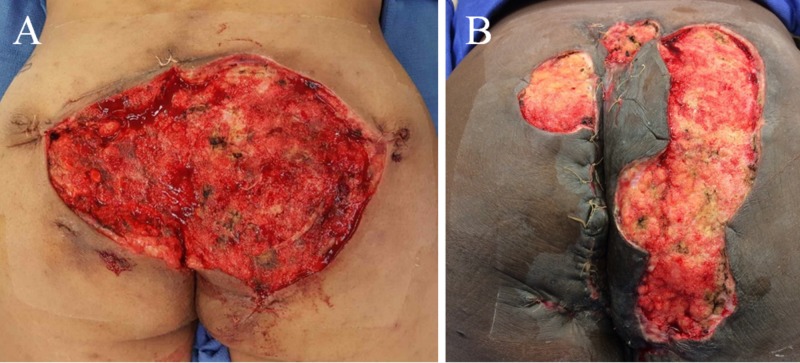
Upon negative pressure wound therapy with instillation and dwell time takedown during the second operation, patients demonstrated healthy pink granulating base without significant fibrinous tissue, slough, or eschar. Patient A was treated for diffuse bilateral buttocks and perianal involvement. Patient B suffered from extensive hidradenitis suppurativa lesions in the left perianal, perineal, and bilateral buttocks regions.

**Figure 2:**
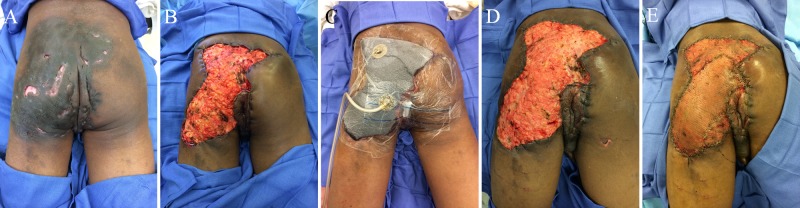
Patient with extensive disease in the left buttock and perineum (A) who underwent wide local excision with tissue advancement (B) and negative pressure wound therapy with instillation and dwell time (NPTWi-d) placement (C); healthy granulating base was found upon NPTWi-d takedown (D) and autologous skin was grafted onto the wound base (E)

All procedures were completed between 2014 and 2017. During the first operation, excision of all diseased skin, abscesses, and fistula tracts was carried down to the level of subcutaneous tissue. Tissue advancement rearrangement was done to minimize the size of the wounds. Vacuum-assisted closure (VAC) VeraFlo™ wound dressing system (NPWTi-d; VAC Instill® Wound Therapy, KCI, an Acelity company, San Antonio, TX) was used to cover the wounds between the initial debridement and definitive closure steps. The instillation agent used was Clorpactin WCS-90 chlorine powder (United-Guardian, Inc., Hauppauge, NY) in one-liter sterile water. The suction was set to -125 mmHg, cycle frequency was set to 3.5 hours, and irrigation dwell time was set to 10 minutes. All patients were admitted to the plastic surgery inpatient service between procedures and placed on bed rest. Patients were brought back to the operating room on postoperative day three to six for split-thickness skin grafting. Upon NPWTi-d takedown during the second operation, all wounds appeared to be in good condition and clean, with robust granulating base and no purulent drainage (Figure 1). Tissue laxity around the wounds allowed additional tissue advancement to further minimize the size of the exposed base and donor site morbidity in some cases. After advancement, tissues were secured with 3-0 absorbable sutures (Vicryl®, Ethicon, Sommerville, NJ). Skin grafts were harvested at 1/12,000 inch from the thighs, meshed to 1.5:1, and secured over the wounds with 3-0 chromic sutures (Ethicon, Sommerville, NJ) (Figure 2). Silver foam dressing and NPWT without irrigation (VAC ULTA™, San Antonio, TX) were placed over the grafts at -125 mmHg continuous suction. Donor sites were covered with Aquacel® (Convatec, Oklahoma City, OK), Tegaderm™ (3M, St. Paul, MN), and elastic bandage.

## Results

**Figure 3:**
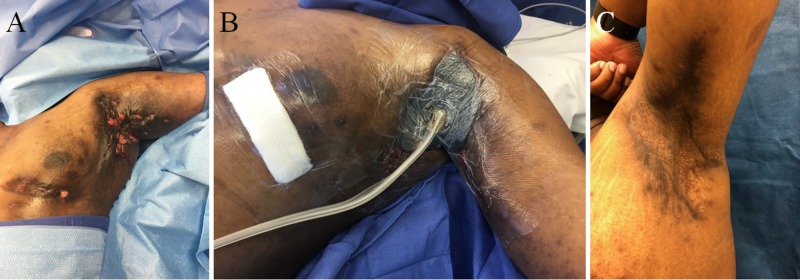
Patient with severe bilateral axillary disease with left chest involvement (A) who underwent local excision, tissue advancement, and negative pressure wound therapy with instillation and dwell time (NPTWi-d) placement (B); postoperative 21 months with no local disease recurrence following successful autologous skin grafting (C)

NPWTi-d did not need to be changed between surgeries and the total number of surgeries for each patient was two. First for excision and NPWTi-d placement and second for the NPWTi-d takedown and skin grafting (Table 1). The volume of irrigant varied between 22-60 ml depending on the size of the wounds. The duration of NPTWi-d placement varied between three to four days with an average of 3.5 days. The interval from excision to complete wound coverage with grafting varied between three to six days with an average of 4.3 days. One patient received skin grafting six days after the initial surgery due to scheduling difficulties; however, his NPWTi-d was removed on postoperative day three and the wound was managed with wet to dry dressing changes until the second procedure. The original wound size measured between 210-540 cm2, skin grafts of similar sizes were harvested from donor sites, and the percent of graft uptake ranged from 70%-90%. The average time from skin grafting to complete healing ranged from eight to 17 weeks. NPWTi-d failure, wound infections, and surgical complications were not observed in any of the patients. None of the patients required repeat hospitalization, reoperation, or regrafting. Patients were followed up to four years postoperatively and all were doing well with no recurrence of local disease (Figure 3).

**Table 1:** Summary of patient outcomes

Patients	I	II	III	IV
Age	34	28	61	51
Sex	M	M	M	M
Body mass index (kg/m^2^)	24.3	33.1	19.3	36.7
Location of disease	Bilateral buttocks, perianal	Bilateral axilla, left chest	Left buttock, perineum	Left perianal, perineal, bilateral buttocks
Area of disease (cm^2^)	391	246	540	210
Vacuum Assisted Closure settings (mmHg, hr)	-125, 3.5	-125, 3.5	-125, 3.5	-125, 3.5
Dwell time (min)	10	10	10	10
Instillation volume (mL)	36	22	22	60
Duration of Vacuum Assisted Closure placement (d)	4	3	4	3
Duration between surgeries (d)	4	6	4	3
Time to complete healing (w)	14	13	8	17
Percent graft uptake	80%	90%	90%	70%

## Discussion

HS is a debilitating disease with physical and psychosocial limitations, made worse by severe wound contamination that slows healing and limits therapeutic options. To date, severe HS continues to be a debilitating condition that remains difficult to treat. Biofilms can be observed in the sinus tracts of 67% of chronic HS lesions and contributes to the difficult nature of the disease [9]. Topical therapies such as clindamycin ointment have been used in patients with superficial HS, but such treatment displays poor efficacy for deeper lesions and abscesses [10]. Systemic antibiotics such as clindamycin/rifampin, metronidazole, and ertapenem have also been used to treat HS and demonstrated variable response and relapse rates [11-12]. Studies utilizing oral retinoids, methotrexate, and dapsone have determined them ineffective as first-line therapies and should only be used in combination with other therapeutic agents [13-15]. Intralesional steroid injections have successfully reduced inflammation in acute HS flares and systemic steroids have shown satisfying results in severe or refractory cases, but are not recommended for long-term use [16-17]. Biologic agents, such as the TNFα antagonists infliximab and adalimumab, have shown promising results in reducing the severity and frequency of recurrences in patients with severe or refractory HS, but are associated with serious adverse events [18-20]. Thus, a multidisciplinary therapeutic approach with combined medical and surgical management has remained necessary for many patients with severe or diffuse HS, but introduces immense wound closure and healing challenges.

NPTWi-d therapy has been demonstrated as one of the most effective methods in assisting wound closure. In addition to improving wound granulation and vascularization, the use of NPWTi-d in the management of complex wounds significantly reduces wound contamination by continuous removal of bacteria and disruption of bacterial attachment to tissues. This results in a reduced bacterial load in wounds complicated by virulent pathogens, and even wounds that harbor pathogens with attachment through biofilms or multidrug resistance [21-22]. In in vivo and in vitro wound models, NPWTi-d has proven superior to NPWT alone through reduction of bioburden in artificially infected wounds [23-24]. In human studies, NPWTi-d has shown to be effective in decontaminating chronically infected wounds in the lower extremities, leading to successful graft uptake even in patients with significant comorbidities [25-26]. While the increased technological complexity of the NPWTi-d system may appear off-putting due to the need for hospitalization for intermittent wound irrigation and increased hospital staffing requirements, NPWTi-d has proven overall advantageous by reducing the number of procedures required for closure in abdominal wounds, hospitalization time, episodes of hospitalization, and total expense of treatment [27].

Instillation solution selection is critical to accomplish cleansing and granulation goals of NPWTi-d. Several solutions can be used with the NPWTi-d system, such as Dakins solution, silver nitrate, normal saline, polyhexanide, and protosan. Successful wound closure has been reported with each of these solutions but the presence of active infection or risk of infection should be taken into consideration when choosing among solutions. Clean wounds can be managed only with saline but contaminated or infected wounds may require an instillation with local antiseptic properties [28]. Clorapactin was chosen due to its powerful germicide, fungicide, and virucide properties. Yet it is nontoxic and nonirritating when used in therapeutic dosages. It is an excellent oxidizing agent and exhibits pronounced wetting, penetrating, and detergent properties. Additionally, it is widely available at a low cost [29].

Other surgical treatment methods for HS include the use of local or perforator flaps and recycled skin grafts [30]. Local and perforator flaps yield successful results in select patient populations, such as patients with small to moderate sized defects. However, both of these methods have significant morbidities and donor site limitations. The excision of advanced HS creates large skin defects, local or perforator flaps may not be adequate for the reconstruction of these defects. The recycled skin graft method was described to decrease the donor site morbidity associated with split thickness skin grafts [30]. The skin over the diseased area is harvested as a split thickness skin graft and reused to cover the defect resulting from the surgical excision of HS. This is a brilliant method that significantly decreases the donor site morbidity if immediate reconstruction is planned. However, NPWTi-d was initially applied to the defects to stimulate granulation formation and reduced bioburden prior to skin grafting in this study. Wounds were covered with skin grafts in a second stage operation. Moreover, reconstructing the defect with the same, chronically infected skin may theoretically increase the risk of postoperative wound infection leading to graft loss.

NPWTi-d is a recent technique successful in other patient populations, most notably orthopedic patients with prosthesis infections [21-22], and may provide an additional option for the management of Hurley stage III HS. This case series offers support for the use of NPWTi-d for severe HS requiring surgical excision and subsequent skin grafting. All patients achieved adequate wound decontamination and tissue granulation within days of excision, shortening the length of their hospitalization. Furthermore, all patients demonstrated successful graft uptake and healed with resolution of local disease. The limitations of our study include: a small sample size; end points based on clinical parameters alone, such as wound appearance, graft uptake, and patient outcome; and that bioburden before and after NPWTi-d was not measured. Additional studies with a larger sample size and microbiological in addition to clinical parameters may be needed to better characterize the use of NPWTi-d in patients with severe and diffuse HS.

## Conclusions

NPWTi-d is a highly successful tool for preparing wounds for closure. It protects the newly developing granulation tissue base and decreases the risk of wound infections. Our study demonstrates that the use of NPWTi-d for the treatment of Hurley stage III HS is not only safe and simple, but also produces excellent outcomes.
